# What Are the Barriers Which Discourage 15-16 Year-Old Girls from Participating in Team Sports and How Can We Overcome Them?

**DOI:** 10.1155/2013/738705

**Published:** 2013-08-29

**Authors:** Abigail R. Wetton, Rebecca Radley, Angela R. Jones, Mark S. Pearce

**Affiliations:** ^1^Department of Sport, Exercise and Rehabilitation, Northumbria University, Newcastle upon Tyne NE1 8ST, UK; ^2^Institute of Health & Society, Newcastle University, Sir James Spence Institute, Royal Victoria Infirmary, Newcastle upon Tyne NE1 4LP, UK; ^3^Human Nutrition Research Centre, Newcastle University, Newcastle upon Tyne NE2 4HH, UK

## Abstract

Given the clear benefits of regular physical activity (such as reduced risks of cardiovascular disease and obesity, as well as other benefits including those related to mental health), exploration of the reasons that adolescent girls give for not taking part in team sports may be particularly valuable for enhancing later rates of participation. We combined questionnaires (*n* = 60) and semistructured interviews (*n* = 6) to assess the barriers that prevent 15-16-year-old girls from participating in extracurricular team games and what can be done to overcome these barriers and improve physical activity levels. Four barriers became prominent as to why girls in this sample do not participate: Internal Factors, Existing Stereotypes, Other Hobbies and Teachers. Methods to overcome these barriers were identified; changing teachers' attitudes and shifting the media's focus away from male sport. Following the successful summer Olympics and Paralympics in the UK, and the resulting positive focus on some of the nation's female athletes, a shift in focus may be possible. However, this needs to be maintained to allow girls more opportunities, role models and motivation to participate in sport.

## 1. Introduction

Habitual physical activity levels and sedentary behaviour in childhood are well established as important to both current and future health of children and adolescents [1, 2]. While studies have reported associations between childhood physical activity and later cardiovascular and metabolic risk markers for adult disease, links also exist between increased physical activity levels in children and greater self-esteem [3, 4] and physical activity later in life [5]. Sport is an obvious way of children to increase their overall physical activity (as one component of physical activity). Participating in team games has also been shown to improve health and quality of life later in life [6]. Studies assessing the contribution of sport to daily energy expenditure are mostly at around 20% [7–9], although a study of Australian adolescents reported contributions up to 40% [10]. Therefore, increasing girls' participation in team sports may be one way of increasing their overall physical activity levels and improving both future health and quality of life.

Sport England has acknowledged that participation in sport in the country needs improvement [11, 12]. Only 16% of the population of England participates in sport three times a week [12], and a half does not participate in any sport each week [11]. Their recent survey states that 25,000 16-year olds drop out of sport each year, suggesting a particular problem in continuing to participate in sport when reaching the end of compulsory education [12]. There is little doubt that younger children do not reach the recommended levels of moderate to vigorous physical activity per day [13–16], so any further drop out later in childhood is worrying. By the age of 15, half as many girls as boys reach the recommended levels of physical activity [17]. It has been suggested that the reason for lower sport participation levels in girls is because of the historical assumption that it is not good for women to participate [18–20]. Modern day females are still faced with some of the same stereotypes, myths, and issues that their foremothers faced [19, 20]. 

In the Sport England Strategy, 2008–2011 [11], a compulsory focus for national governing bodies was to develop females' game. Sports not adhering to this risked the loss of funding, allowing sports that improve girls' participation to be awarded extra funding [11]. Three years after the strategy had begun, participation levels in girls aged 16 were still low (21%) [17]. Given the clear benefits of regular physical activity, of which sport can be a major component, exploration of the reasons that adolescent girls give for withdrawing from sport may be particularly valuable for enhancing rates of participation [21].

In the UK, the emphasis on team sports dominating the physical education (PE) curriculum has favoured boys as preferred sports for girls such as gymnastics and dance were pushed to the side [22]. As sports socialisation in girls is greater in individual sports such as gymnastics and swimming [23], both of which carry a “team” element, the reduction in both team and individual sports that girls prefer suggests a likely overall reduction in sports-based activity in girls of school age. Team games may have “carry over” potential either into adult team game participation, if there is the opportunity, or into other more easily accessible activities; so it is important to determine why girls do not play in team sports and to find ways of overcoming perceived barriers to participation. It has been estimated that if no action was taken regarding physical activity in young people, 50% of women would be obese by 2050 [24]. A new initiative in the UK designed to build on the 2012 summer Olympic and Paralympic games is the creating a sporting habit for life strategy [25]. This is primarily focussing on increasing participation in sports in 14–25-year olds. 

In this explorative study, using mixed quantitative and qualitative methods, the reasons for 15-16-year-old girls not participating in extracurricular team games were investigated, by exploring the barriers they face. Further, possible methods to overcome the barriers were identified. In much previous related research, this has been noticeably absent [26–28], and where present, the research was published at least fifteen years ago [22, 29]. With girls' participation rates decreasing since then it is important to address this issue in contemporary settings. Our aims were to gain a greater understanding of these issues which may help, in the future, to develop interventions to increasing team sports participation in girls.

## 2. Methods

Participants were students at two high schools in the English Midlands. Thirty girls from each school completed a questionnaire designed specifically for this study, based on previous research findings. This was piloted in a group of 5 girls not included in the study. The questionnaire (the appendix) focussed on aspects of participation in team sports, covering availability, views regarding girls' participation and perceived barriers to participation, and setting the background for the qualitative component of the study. Six girls were selected for a short face-to-face semistructured interview (with ARW), following a pilot session with a girl not included in the study. These were girls who stated they did not participate in extracurricular team sports in their questionnaires; offering the opportunity to explore why in more detail. Using a qualitative method as part of the research design allowed a more sensitive approach to investigating the motivational factors influencing the decision to not play sport as the interviews were shaped towards the individual's life and social context [30]. Semi-structured interviews were chosen to gather the research as they allowed participants to express their true feelings in more detail following on from the questionnaires [30]. Interviews were divided into three sections: an introduction, which introduced the interviewer and reviewed the aims of the study, main body, where questions were asked and finally a concluding gratitude sentence, as having different sections splits the interview up and allows the participant to be at ease from the introduction through to the concluding sentence [31].

All interviews were audio recorded and transcribed. Following the evaluation of the descriptive data from the questionnaires, a thematic analysis of the transcripts was used to allow themes and categories to emerge through the use of coding memos which permitted patterns to be identified [32, 33]. Anonymised quotes are used for illustrative purposes, with only the study identifier given to denote which participant gave which quote.

Ethical approval was obtained from the Research Ethics Committee of the Faculty of Life Sciences at Northumbria University. Each student (and a parent) gave her written consent for the student to participate. 

## 3. Results

### 3.1. Quantitative Results

Data from 60 children (42 aged 15, 18 aged 16), 30 from each school, were available for quantitative analysis. The responses to a series of questions related to previously suggested reasons as to why the participants thought that girls did not participate in team games are summarised in Table 1. Although 51 (85%) of the participants did not participate in a team sport, 43 agreed with the statement that their school offered a good range of extra-curricular team games. Feelings about team games were inconsistent, with 30% indicating that they liked team games, and 25% stating that they did not. Girls did not perceive sport as “being a man's game” as a reason for low participation, nor did they agree that “it is not seen as cool for girls to play sport.” However, 55% agreed that their (self-perceived) lack of sporting ability stopped them from participating, and 45% agreed that negative experiences were the causes of not playing team sports outside of school. The lack of enthusiasm and motivation from PE teachers was noted by the majority, who agreed that their teachers focussed on the better players. Similarly, 70% felt that girls' sports teams are not treated equally by their teachers, compared to the boys' sports teams. 

### 3.2. Qualitative Results

From the analysis of the six interviews through coding, several prominent themes emerged (“internal factors,” “existing stereotypes,” “other hobbies,” and “teachers,”) in terms of what prevented girls from participating (i.e., perceived barriers).

#### 3.2.1. Internal Factors

A common theme that was drawn from the participants' data was their perceived lack of ability in sport resulting in a lack of enjoyment. It was noted that embarrassment, pressure, or a self-perceived lack of ability in PE lessons directly influenced girls. ID5 stated “The teacher embarrassed me and my friend in front of everyone…makingus feel lower.” Further responses indicated that experiences like these resulted in rejecting extracurricular sports as self-confidence and self-belief had been damaged. ID6 stated “People are too competitive in them [Team Games]…I'm one of the bad people so I don't want to mess it up for the good people.”

#### 3.2.2. Existing Stereotypes

Participants showed recognition of the stereotypes about girls playing sport. ID2 stated “The stereotype will be that boys will always be better than girls because that's what people think.” All participants noted that stereotypes were predominantly created through the media, and that they were not aware of any high profile female sports stars that they could aspire to. ID5 agreed that the media have cemented masculinity within sport, through their stereotype presentation of female athletes: “Sport is seen as a manly thing to do…they [the media] don't see it as a girly thing.” Further findings suggested the family background of the girls influenced their decision to not participate; as their parents offered similar stereotypical views to the media.

#### 3.2.3. Other Hobbies and Time Commitments

The desire to partake in other activities, as an alternative to playing sport or as a preference in terms of using available free time, was common. ID4 remarked on how her other interests restricted her in staying after school: “I cook a lot…so can't find the time…don't really want to do it.” ID5 reiterated this, as she emphasised the fact that other pastimes have precedence over sports: “Don't have the time…have other hobbies that I would rather do like arty things.” ID2 stressed the lack of time available because of being in the final year of standard secondary school with GCSE examinations looming “I never have the time…when you're in year eleven you have GCSE's and loads of coursework.”

#### 3.2.4. Teachers

All of the interviewed participants highlighted teachers as having a role in their lack of participation. ID3 stressed the lack of attention the teachers gave her: “People who are better, they praise them more, with people who are not very good they just criticise them constantly by having a go at them.” The remainder of the participants expressed their equally negative opinions; firstly, boys' teams received higher praise than girls' teams for their achievements and secondly the teachers already knew who they wanted in their teams, which consequently led to a lack of information being delivered regarding training sessions. 

### 3.3. Methods to Overcome Barriers

Two strong themes ensued. Firstly; all participants agreed that media influences, such as newspapers and the television, must reduce their favouritism towards male sports. ID2 believed that: “The media only ever talk about male sports, like football; if you read a magazine or a newspaper you will never ever see a girls' team in there.” Secondly, all participants noted the perceived negativity of their teachers; hence the importance of changing the attitudes of teachers became clear throughout. ID2 suggested “…We should be given a choice of what sports you can play in lessons, so you will want to play them after school as well.” ID3 commented on the lack of information provided by the teachers stating that: “Teachers should give more notice about clubs.” One final matter concerning teachers was that participants reported that less competition would encourage more girls to play in team games. ID1 recommended “Teachers should form different teams…all schools should have second, maybe third teams just so if girls aren't good enough for the first team they can still play.” This was echoed by ID5 who added “If they had a higher level group and a middle level group…that would be good, then it would be something extra to do with your friends.”

## 4. Discussion

In this mixed methods study of 15-16-year-old girls in The Midlands region of England, a number of barriers to their participation in team sports were identified, as were potential routes to overcoming these barriers. The barriers fall into four main themes: (i) internal factors such as confidence, pressure, embarrassment, or negative self-belief; (ii) existing stereotypes and the lack of female role models; (iii) other hobbies or commitments that result in a lack of time or preference for sport; and (iv) teachers and their perceived concentration on boys' teams or the girls with high levels of sporting ability. In terms of overcoming these barriers, there were two clear themes: (i) the media and a need for better representation of female sports and female athletes as role models, and (ii) the need for teachers to provide more encouragement and opportunities for girls to participate, regardless of their ability.

The findings further support those in previous studies suggesting that a number of barriers are presented against girls participating in team sports [21, 22, 34, 35]. Our findings regarding internal factors are consistent with previous evidence showing that females were reluctant to participate in extracurricular sports, as they tended to display less confidence in their physical ability [36, 37]. The findings from the questionnaire compliment this theme as over half agreed that this factor halts their inclusion. With similar findings from both sources of data, it suggests that personal ability, or at least the lack of self-belief in their ability, contributes to why girls do not participate in team sports. The findings also suggest that team games are avoided by girls because of the competitive nature they bring with them. It may also be the case that girls do enjoy team games; however their ability halts their involvement as the teams available at school represent the elite ability of their peers. If teachers created teams that placed girls with lower ability together, this may allow them to participate freely without becoming anxious; suggesting teachers' attitudes towards girls and sport may be more relevant than personal ability. This may be possible in the UK through the new “Creating a sporting habit for life strategy” [25], which includes a push for young people of different abilities to be able to participate in competitive sports. This is both within educational institutions, but also includes a move to improve and build on existing links between schools and community sports clubs to enable young people to continue to participate after leaving education. However, there is also an issue of the competitive nature of team sports. Creating additional teams where enjoyment is the preferred outcome could potentially allow girls to feel less pressured when playing, as some girls are discouraged by the competitive nature team sports can bring [38]. As the “Creating a sporting habit for life strategy” has a large focus on competitive sport [25], this strategy may work better for boys than girls, given our findings.

Participants recognised the stereotypes about girls playing sport, as seen in previous studies [19–21], and all viewed that stereotypes were predominantly created through the media, with a lack of high profile female sports stars that they can aspire to. From these findings, it demonstrates that stereotypes still exist within not only society but also crucially within schools. Images of male teams fill the most coveted magazines and newspapers, creating assumptions that girls do not fit in unless they support stereotypical masculine traits [39]. Further findings suggested the family background of the girls influenced their decision to not participate; as their parents offered similar stereotypical views to the media. However, the quantitative results suggested this was not a collective theme for girls participation; as when asked whether it was not seen as cool for girls to play sport, almost half disagreed, meaning stereotypes may only be a factor for certain girls rather than all girls not playing extracurricular team sports. 

Both the questionnaire and interviews supported the concept of other hobbies or commitments taking priority over team sports, consistent with previous research [21, 35]. However, Biddle et al. previously suggested that, although girls may be completing other activities, there is an underlying explanation for this choice, that girls, from an early age, have been socialised into believing sports are not in their nature [27]. Therefore it is difficult to determine from this study whether the participants had been socialised to not play extracurricular team games beforehand, thus resulting in their time preferences.

The perceived negative influence of teachers is consistent with other studies that stated that PE teachers contribute to girls excluding themselves from extracurricular team sports [15, 26–29]. In the study by Jago et al., one participant mentioned how her PE teacher restricted her inclusion in football by telling her “we don't do football, so you can't do it;” the participant then described how she had refused to join in PE lessons since [15]. Likewise, ten years earlier, Penney reported that PE teachers lacked the knowledge and beliefs of what girls wanted to do in PE and after school clubs, with one teacher stating “I don't think football should be taught to girls” [28]. 

Our findings in terms how to overcome these barriers are primarily based on two of the barrier themes: stereotypes (in the form of the media) and the influence of teachers. The findings support the assertions of earlier theorists, who argued that women in sport are belittled by the media, being continuously marginalised and trivialised [22, 34]. It is apparent that from our findings and previous research that, on a larger scale, one method is to publicise female sport through the media, giving school girls the role models they require [40–42]. However; it is questionable whether the media would ever change the male dominated structure, as Daniels found that even in mainstream magazines for teenage girls, female athletes are largely invisible [39]. If girls' magazines are not willing to promote sport, it seems highly unlikely that male-dominated newspapers and television programmes will follow suit [39], suggesting this method requires wholesale change. The wide and positive coverage given to successful female Olympians and Paralympians was noticeable in the UK during the 2012 summer Olympics and Paralympics, but it remains to be seen if this was simply a temporary situation for the UK media.

With regard to the influence of teacher and a need for changes in the way that schools handle team sports for girls, these findings are consistent with previous suggestions that for girls to participate in extracurricular team sports, the teachers need to be motivators [22, 28]. Enthusiastic teachers should, in turn, encourage less confident girls to try sports, even new sports that may appeal to the lower ability cohort [22, 28]. The task of changing teacher attitudes is not a straightforward one; Williams and Bedward [43] previously argued that powerfully stereotypical attitudes remained among PE teachers, consequently resulting in teachers refusing to alter their beliefs, for example, still not allowing girls to play “masculine” sports in physical education. Cardon et al. recently found that the number of teachers willing to guide after-school activities is low, making it difficult to predict whether teachers would want to start new clubs for girls [44]. 

This study was undertaken in one region of the country, using only two schools, with sixty participants in total. This limited the number of different perspectives on certain themes such as teachers and available sports clubs. While different results may have been found using schools in different areas, the consistency with much of the previous literature suggests that little bias has been introduced. We did not ascertain whether any of the girls not participating in team sports were previously experiencing team sports and had dropped out, although previous experiences are likely to have been incorporated into the qualitative component of the study. Whether the participants had low overall physical activity levels or not was not determined during this study, so it cannot be disproven that physical activity levels are the cause or effect of the themes discussed, due to the potential for bidirectional associations between physical activity levels and other factors [16]. Further research would be able to test this, investigating whether low physical activity levels were a cause or an effect of themes from this study. However, in terms of identifying barriers to participation, the themes identified in this study corresponded with those identified earlier from previous research [15, 22, 28]. Although the teachers were a perceived barrier, the gender of the PE teachers was not available; therefore further research could involve examining whether the gender of teachers influences girls' participation. It would also be useful for a similar study of teachers to see whether they agree with the thoughts of their pupils and what they perceive to be the barriers to implementing the changes required to increase participation in team sports among girls. It would also be of interest to have a similar qualitative component addressing why the girls who do participate in team sports do so. However, the number of girls in this group was too small in this study to form an adequately-sized pool of potential participants. It may also be useful for a future study to also include boys to assess whether similar perceptions and barriers exist.

## 5. Conclusions

Four barriers were identified as to why girls aged 15 and 16 years in this sample do not participate in extracurricular team games: Internal Factors, Existing Stereotypes, Other Hobbies, and The Teachers. Following these themes, methods to overcome these barriers were also identified, changing teachers' attitudes and shifting the media's focus away from male sports; suggesting two different scales of change. Altering teachers' ideologies is a small-scale tactic within schools, but would require large-scale changes of teaching ideologies and policies in many schools to have a wider effect. Directing the media into female organised and competitive sports requires wholesale change, on a national scale, which would be difficult to implement without a wider shift in both media and educational and sports governing bodies policies as well as societal views regarding female sports and sportswomen. It is to be hoped that by following the successful summer Olympics and Paralympics in the UK and the resulting positive focus on some of the nation's female athletes a shift in focus may be possible. However, this needs to be maintained to allow girls more opportunities, role models, and motivation to participate in sport.

## Figures and Tables

**Table 1:** Girls' views as to why girls do not take part in team sports.

Reason	Strongly agree	Agree	Neutral	Disagree	Strongly disagree
Teachers focussed on good players	26	26	3	4	1
Negative experiences from PE lessons	8	27	17	7	1
Lack of personal ability	7	33	10	9	1
Not seen as cool for girls to play sport	3	11	8	29	9
Girls should not play sport because it is a “Man's game”	3	8	6	16	27
Other personal hobbies	9	34	15	1	1

## Appendix

### The Questionnaire Used in the Quantitative Part of the Study

1. How old are you? (Please circle appropriate answer)
  15
  16
2. Does your school offer PE as a GCSE? (Please circle appropriate answer)
  Yes
  No
3. If yes, did you choose PE as one of your GCSE'S? (Please circle appropriate answer)
  Yes
  No
4. What team sports are available for girls to participate in at your school? (List the sports in the space below)
5. ……………………………………………
6. Do you participate in any extracurricular sports clubs? (Please circle appropriate answer)
  Yes
  No
7. Do you prefer to participate in team sports or individual sports? (Please circle appropriate answer)
  Team Sports
  Individual Sports
  Neither
8. On a scale of 1–5 how do you feel about participating in team sports (Circle answer)
  1 (I hate them)
  2
  3
  4
  5 (I love them)
9. Do you participate in a team sport for your school? (If no go straight to number (11))
  Yes
  No
10. …if so, which sport(s) (Write answer below)
11. ……………………………………
12. Why did you choose to participate? (Write answer below, and go straight to question (12))
13. ……………………………………
14. Why do you not participate in a team sport?
15. ……………………………………
16. There is a good range of extracurriculum sports available at school (Circle most appropriate statement)
  □ Strongly Agree
  □ Agree
  □ Neither Agree or Disagree
  □ Disagree
  □ Strongly Disagree
17. Previous Studies have suggested the following as the reasons girls do not participate in team games. Tick the box you agree with for each option
18. Teachers focused on good players
  □ Strongly Agree
  □ Agree
  □ Neither Agree
  □ Disagree
  □ Strongly Disagree
19. Negative experiences from PE lessons
  □ Strongly Agree
  □ Agree
  □ Neither Agree
  □ Disagree
  □ Strongly Disagree
20. Lack of Personal Ability
  □ Strongly Agree
  □ Agree
  □ Neither Agree
  □ Disagree
  □ Strongly Disagree
21. Not seen as cool for girls to play sport
  □ Strongly Agree
  □ Agree
  □ Neither Agree
  □ Disagree
  □ Strongly Disagree
22. Girls should not play sport because it is a “Man's game”
  □ Strongly Agree
  □ Agree
  □ Neither Agree
  □ Disagree
  □ Strongly Disagree
23. Other Personal Hobbies
  □ Strongly Agree
  □ Agree
  □ Neither Agree
  □ Disagree
  □ Strongly Disagree
24. Please state any other reasons you believe discourage or prevent girls your age from participating in team sports
25. ………………………………………
26. (A) Do you feel boys and girls sports teams are treated equally at school by the teachers? (Circle answer)
  Yes
  No
27. (B) Please explain your answer (Write answer in space provided)
28. ………………………………………
